# Deficiency of the *Tmem232* Gene Causes Male Infertility with Morphological Abnormalities of the Sperm Flagellum in Mice

**DOI:** 10.3390/cells12121614

**Published:** 2023-06-13

**Authors:** Xiuqing He, Wenyu Mu, Ziqi Wang, Ke Xu, Yingying Yin, Gang Lu, Wai-Yee Chan, Hongbin Liu, Yue Lv, Shangming Liu

**Affiliations:** 1 School of Basic Medical Sciences, Shandong University, Jinan 250012, China; hexiuqing2022@163.com; 2 Center for Reproductive Medicine, Shandong University, Jinan 250012, China; 17853137722@163.com (W.M.); wangziqi1110@126.com (Z.W.); 202020745@mail.sdu.edu.cn (K.X.); 18865381921@163.com (Y.Y.); hongbin_sduivf@aliyun.com (H.L.); 3 Key Laboratory of Reproductive Endocrinology of Ministry of Education, Shandong University, Jinan 250012, China; 4 Shandong Provincial Clinical Medicine Research Center for Reproductive Health, Shandong University, Jinan 250012, China; 5 Shandong Technology Innovation Center for Reproductive Health, Jinan 250012, China; 6 CUHK-SDU Joint Laboratory on Reproductive Genetics, School of Biomedical Sciences, The Chinese University of Hong Kong, Hong Kong, China; lugang@cuhk.edu.hk (G.L.); chanwy@cuhk.edu.hk (W.-Y.C.); 7 Shandong Key Laboratory of Reproductive Medicine, Shandong First Medical University, Jinan 250117, China

**Keywords:** TMEM232, ODF1, sperm, axoneme, cytoplasm removal

## Abstract

The axoneme and accessory structures of flagella are critical for sperm motility and male fertilization. Sperm production needs precise and highly ordered gene expression to initiate and sustain the many cellular processes that result in mature spermatozoa. Here, we identified a testis enriched gene transmembrane protein 232 (*Tmem232*), which is essential for the structural integrity of the spermatozoa flagella axoneme. *Tmem232* knockout mice were generated for in vivo analyses of its functions in spermatogenesis. Phenotypic analysis showed that deletion of *Tmem232* in mice causes male-specific infertility. Transmission electron microscopy together with scanning electron microscopy were applied to analyze the spermatozoa flagella and it was observed that the lack of TMEM232 caused failure of the cytoplasm removal and the absence of the 7th outer microtubule doublet with its corresponding outer dense fiber (ODF). Co-IP assays further identified that TMEM232 interacts with ODF family protein ODF1, which is essential to maintain sperm motility. In conclusion, our findings indicate that TMEM232 is a critical protein for male fertility and sperm motility by regulating sperm cytoplasm removal and maintaining axoneme integrity.

## 1. Introduction

In mammals, male fertility is determined by the ability to successfully produce motile sperm carrying the complete paternal genome and having the ability to fertilize the oocyte. Spermatozoa are generated via a complex process, i.e., spermatogenesis, which is categorized into three primary steps, namely, mitosis (spermatogonia proliferation), meiosis (decrease in the chromosome numbers from diploid to haploid) as well as spermatogenesis (differentiation of spermatids into spermatozoa) [1]. In the course of spermatogenesis, round spermatids experience a complex series of cytological events which transform them into mature spermatozoa. These events consist of (i) nuclear condensation, (ii) acrosome formation, (iii) flagellum formation and (iv) finally, the spermatid releases large amounts of cytoplasm as remnants that are engulfed by spermatogenic cells [1,2].

Spermatozoa are highly specialized cells which comprise a head region and flagella. The head region carries the paternal genome and the flagella is essential for the motility of spermatozoa [2]. The flagella of mammalian sperm can be classified into four segments: the connecting part joining the head and the middle part consisting of mitochondria closely arranged around the cytoskeletal structure of the flagella, with the major part accounting for about 2/3 of the sperm flagellum length and the end part being shorter [3]. The main cytoskeletal structures of the flagellum are the axonemes, which consist of a central pair of microtubules (CP) that are surrounded by 9 external microtubule doublets (MTDs) in a “9 + 2” pattern [4]. The flagellum of spermatozoa has distinctive accessory components surrounding the central axoneme in different segments. In the midpiece, the central axoneme is enclosed by outer dense fiber (ODF) together with a mitochondrial sheath (MS) while in the major part, it consists of ODFs and fibrous sheaths (FS) [4,5,6,7]. Prior studies have demonstrated that ODFs protect the sperm tail from shear forces by preserving flagellar elasticity throughout epididymal transport and ejaculation. To date, four primary proteins have been discovered in the ODFs, named the ODF family proteins: ODF1, ODF2, ODF3, and ODF4 [3]. Previous research has supported the notion that the ODFs’ structural integrity enhances sperm motility via modulating the stability of axonemes [8].

Several transmembrane protein family proteins have been found to play essential roles in sperm motility and sperm–oocyte fusion. Studies on the transmembrane protein TMEM16E discovered that it is expressed in the germ cells during early spermatogenesis and in mature spermatozoa and was noted to be localized specifically to the tail of spermatozoa. Spermatozoa of *Tmem16e* knockouts (KOs) exhibited decreased motility and poor fertilization efficiency with cumulus-free but zona-intact oocytes in vitro [9]. These findings demonstrate that TMEM16E acts as a phospholipid scramblase within inner membranes, and dysfunction in TMEM16E significantly impacts sperm motility [9]. TMEM190 also falls into the family of transmembrane proteins and has specific expression in the mouse testis. Immunoprecipitation experiments showed that TMEM190 protein is a component of the mouse sperm protein complex, which acts an indirect player in the fusion of sperm and oocytes [10]. TMEM95 is also a highly expressed transmembrane protein in mice testes that is pivotal for the interaction of sperm with oocytes, and male *Tmem95* KO mice are sterile because of impaired fusion of sperm with oocytes [11]. In the screening and investigation of mouse testis-enriched genes, we detected transmembrane protein family 232 (*Tmem232*) gene, which has high expression in the testis. *Tmem232* KO mice were produced to characterize the functions of TMEM232 in mice. We reveal that *Tmem232* KO male mice are sterile, and this work also provides evidence that *Tmem232* dysregulation can influence the stability of axonemal structures and cytoplasmic clearance during spermatogenesis.

## 2. Materials and Methods

### 2.1. Animals

The mouse *Tmem232* gene (Transcript: ENSMUST00000062161) is 284.77 kb and encompasses 16 exons, and is positioned on chromosome 17. For generating *Tmem232* knockout mice, a specific target region from exon 4 to exon 8 was selected. The CRISPR-Cas9 system (Cyagen Biosciences, Suzhou, China) was employed to accomplish this. Co-injection of Cas9 and gRNA mRNA into the fertilized eggs of C57BL/6 mice produced a target line with a 21,844 bp deletion. Deletion in the founder animals was confirmed with genomic DNA sequencing. All experiments on animals were carried out as per the procedures approved by the Animal Use Committee of the School of Medicine, Shandong University.

### 2.2. Plasmids

Mouse *Tmem232* and *Odf1* were cloned into the pCMV-HA and pCMV-Flag vectors, respectively. The two plasmids were generated by miaolingbio. Inc., Wuhan, China.

### 2.3. Antibodies

Rabbit anti-ODF2 antibody (1:1000 dilution, 12058-1-AP, Proteintech, Rosemont, IL, USA) and mouse anti-α-tubulin antibody (1:1000 dilution, F2168, Sigma-Aldrich, St. Louis, MI, USA) were used for the immunofluorescence assays. Mouse anti-FLAG antibody (1:1000 dilution, TA50011-100, Origene, Rockville, MD, USA) and rabbit anti-HA antibody (1:1000 dilution, 3724S, Cell signaling technology, Danvers, MA, USA) were applied for the Western blotting.

### 2.4. Immunoprecipitation

HEK293T cells that were transfected with the target constructs were lysed using cell lysis buffer for Western blot and IP (Poo13, Beyotime, Shanghai, China) supplemented with 1 mM PMSF. The cell lysates were incubated on ice for 30 min, and later centrifuged at 12,000× *g* for 30 min. The cell lysates were incubated with anti-FLAG antibody for immunoprecipitation at a temperature of 4 °C overnight. Subsequently, Protein A/G Agarose Beads (PROTAA-RO, PROTGA-RO, Merck, KGaA, Darmstadt, Germany) were added to the lysates and incubated at 4 °C for 60 min. The precipitate obtained was cleaned three times utilizing cell lysis buffer, and the immune complexes were eluted through incubation with the sample buffer with 1% SDS at a temperature of 95 °C for 5 min. Lastly, the samples after elution were analyzed by immunoblotting [12].

### 2.5. Immunoblotting

The extracted proteins from the immunoprecipitates or lysates were isolated by SDS-PAGE and transferred to nitrocellulose membranes. Subsequently, membranes were blocked with 5% skim milk (BD, 232100, Franklin Lakes, NJ, USA) and were incubated with the specific primary antibodies. Primary antibodies were examined utilizing Alexa Fluor 800- or 680-conjugated goat anti-mouse or goat anti-rabbit secondary antibodies. The scanning and visualization of the results were performed via exploiting the Tanon-5200 Chemiluminescent Imaging System (Tanon Science & Technology, Shanghai, China) [13].

### 2.6. Immunofluorescence in Testes

Testes from *Tmem232* and WT KO mice were fixed with 4% PFA at a temperature of 4 °C overnight. The testes were dehydrated in 70% ethanol (*v*/*v*) after fixation and later embedded in the paraffin. Histological analysis was implemented on glass slides mounted with 5 µm sections. Subsequently, the slides were deparaffinized and then rehydrated. Antigen retrieval was implemented using 1× Sodium Citrate Antigen Retrieval Buffer (with a pH of 6.0) (PR30001, Proteintech) for 30 min, and then washed with PBS 3 times (with a pH 7.4). To prevent nonspecific binding, the slides were blocked using a solution of 0.1% Triton X-100 and 5% BSA and incubated with the primary antibody at 4 °C overnight. Afterwards, the slides were washed a minimum of 3 times with PBS and incubated with Alexa Fluor 594- or 488-conjugated secondary antibodies for an hour at ambient temperature. Cell nuclei were stained using DAPI, and the images were acquired with an SP8 microscope (Leica, Wetzlar, Germany) [14].

### 2.7. Immunofluorescence in Single Spermatozoa

The mouse epididymal sperm samples were collected and prepared by spreading onto glass slides. The slides were air dried and maintained at −80 °C for follow-up immunofluorescence tests. The glass slides with the spermatozoa were fixed with 4% PFA for 15 min at RT and washed a minimum of three times with PBS. Then, the slides were blocked with 5% BSA at RT for 30 min. After blocking, the slides were incubated with primary antibody at 4 °C overnight. Following this, the slides were washed a minimum of 3 times with PBS and incubated with secondary antibodies conjugated with Alexa Fluor 594 or 488 for an hour at RT. The slides were cleaned completely using PBS to remove any residual secondary antibodies, and then later stained using DAPI. Lastly, images were taken using an SP8 microscope (Leica) [13].

### 2.8. Real-Time PCR

For the RT-PCR assays, the total RNA of multiple adult tissues and 8–56-day-old mouse testes was isolated from C57BL6/J hybrid mice. cDNA was prepared using the HiScript Q RT SuperMix for qPCR (Vazyme, Nanjing, China) as per the instructions of the manufacturer. PCR was implemented with SYBR Green Pro Taq HS Premix Taq (Accurate Biology). All RT-PCR reactions followed the same protocol, first with an initial denaturation step at 95 °C for 10 min. Then, denaturation at 95 °C for 30 s, annealing at 60 °C for 30 s, and extension at 72 °C for 45 s for 30 cycles. The last extension step was carried out at 72 °C for 7 min utilizing a (Bio-Rad) T100 Thermal Cycler. The primers employed for amplification were as follows: Forward: 5-AGTGCAAAGGGGAGATCCAAA-3 and Reverse: 5-GGTTGACTTCGTAGCCCCTC-3, generating a 572 bp fragment. The housekeeping gene *Gapdh* was amplified utilizing the following primers: Forward: 5-GCCTTCTCCATGGTGGTGAA-3 and Reverse: -GCACAGTCAAGGCCGA GAAT-3 [13].

### 2.9. Tissue Collection and Histological Analysis

Caudal epididymis and testes were isolated from a minimum of 3 *Tmem232* KO and 3 WT mice. The samples were dissected immediately after euthanasia and fixed for up to 24 h in 4% (mass/volume) paraformaldehyde (Solarbio, Beijing, China, P1110). Afterwards, the samples were dehydrated in 70% (*v*/*v*) ethanol and later embedded in paraffin. In order to conduct a histological analysis, sections (5 µm) were created and then mounted on glass slides. The sections were subsequently stained using PAS and H&E [12,13].

### 2.10. Mouse Sperm Collection

We dissected the caudal epididymis, including the 4–5 cm proximal vas deferens, from at least three WT and three *Tmem232* KO mice and washed them in phosphate-buffered saline (PBS). The caudal epididymides, including the 4–5 cm proximal vas deferens were dissected from at least three WT and three *Tmem232* KO mice and washed in phosphate-buffered saline (PBS). The vas deferens and caudal epididymis of each animal were subjected to sperm recovery through flotation. The vas deferens and caudal epididymis were incised using a scalpel in a Petri dish with PBS (1 mL). After 30 min in a 5% CO_2_ atmosphere at 37 °C, the tissue was removed and the suspension of sperm was assessed. The sperm suspension was used for sperm counting, morphological analysis and immunofluorescence experiments [13,15].

### 2.11. Sperm Motility Assessment Using CASA

Sperm motility was analyzed in sperm samples from a minimum of 3 *Tmem232* KO and 3 WT mice with an Olympus B× 51 microscope (Olympus, Tokyo, Japan) using a 20× phase objective. During analysis, a sperm sample (10 µL) was put into the glass cell chamber (Hamilton Thorne, Beverly, MA, USA, 80 µm 2X-CEl) at an 80 µm depth. A CCD camera was used to acquire images, which were analyzed utilizing the Animal Motility system (Hamilton Thorne, Beverly, MA, USA). Parameters, i.e., progressive spermatozoa, total motility, straight-line velocity as well as average path velocity, were evaluated on the basis of the analysis at least 200 spermatozoa per sample. Semen analysis was repeated a minimum of 3 times [12].

### 2.12. Mouse Fertility Testing

Mature female and male mice (between 6 and 8 weeks of age) were paired together in successive mating trials. The number of litters and date were recorded, and the pups produced were moved away from the breeding pair so that the pair continued mating up to the end of experiment [16]. For testing the fertility of males in mice, individual males (KO and WT) were mated with WT females for six months, and the pup numbers produced by each female were recorded in this experiment. Likewise, to assess female fertility, the individual wild-type male mice were mated with both KO and WT females for six months, and the number of litters produced by each female in this experiment was recorded.

### 2.13. Transmission Electron Microscopy

The caudal epididymis together with testes were dissected from a minimum of three WT and three *Tmem232* KO mice. The samples were pre-fixed overnight at a temperature of 4 °C with 2.5% (*v*/*v*) glutaraldehyde in theobromine buffer (0.1 mol/L). After pre-fixation, the samples were washed using cacodylate buffer (0.1 mol/L) and later cut into small pieces about 1 mm^3^ in size. Afterwards, they were soaked in 1% OsO_4_ at 4 °C for an hour. The samples were dehydrated with an array of graded acetone solutions and later embedded into resin (Low Viscosity Embedding Media Spurr’s Kit, EMS, 14300) for staining. After cutting into ultra-thin sections using an ultramicrotome, the sections were double stained with lead citrate along with uranyl acetate. Images of the stained sections were acquired using a JEM-1400 transmission electron microscope [17], and subsequent analysis was performed.

### 2.14. Scanning Electron Microscopy (SEM)

The caudal epididymis was dissected from a minimum of 3 *Tmem232* KO and 3 WT mice. The extraction of sperm was conducted via squeezing the caudal epididymis, and the semen samples were fixed on coverslips using 2.5% glutaraldehyde. Subsequently, the samples were further fixed with osmium tetroxide. After the samples were washed 3 times with distilled water, the sperm samples were dehydrated with a series of concentrations of cold ethanol (namely, 50%, 70%, 95% and 100%). After dehydration, the samples were dried with a Tousimis Autosamdri-810 Critical Point Dryer and mounted on a specimen base. Prior to imaging and analysis, the coverslips were sputtered on a co-rail clamp, which was conducted using a FEI Quanta ESEM 200 SEM [17].

### 2.15. Statistical Analysis

All tests were carried out with a minimum of three repetitions, and the results are presented as mean ± SEM. The statistical analysis was implemented through applying the Student’s *t*-test with a paired two-tailed distribution and with two-way ANOVA test in GraphPad Prism 6 (GraphPad). Differences between means of various genotypes were deemed to be statistically significant at *p*-values < 0.05.

## 3. Results

### 3.1. TMEM232 Is an Evolutionarily Conserved Testis-Enriched Protein

The NCBI database Basic Local Alignment Search Tool (BLAST) (BLAST: Basic Local Alignment Search Tool (nih.gov)(accessed on: 24 December 2022)) shows that TMEM232 is a highly conserved protein in mammals (mouse, chimpanzee, rat, human, cattle and dog) (Figure S1A). To profile *Tmem232* mRNA expression, we conducted RT-PCR utilizing multiple organs of adult mice. *Tmem232* has high expression in the testis and weak expression levels in the spleen, liver, brain, uterus, lung, epididymis and kidney; however, it was not detected in the heart or ovary (Figure S1B). The analysis began with testis samples from the early postnatal period (to capture the frontier of the first wave of spermatogenesis) which showed that the mRNA expression of *Tmem232* was raised markedly around post-natal day 21 (Figure S1B), which is roughly equivalent to the haploid and round sperm stage of spermatogenesis [18]. These results indicate that *Tmem232* is a gene that is evolutionarily conserved and enriched in the testis.

### 3.2. Tmem232 Knockout Male Mice Are Infertile

**Figure 1:**
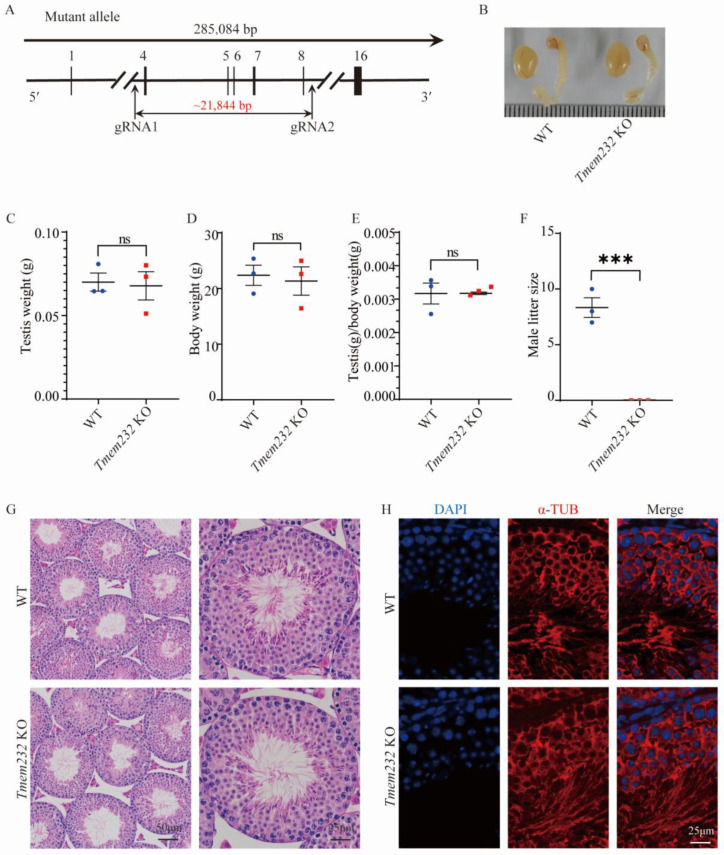
The knockout of *Tmem232* causes infertility in male mice. (**A**) The CRISPR-Cas9 strategy for generating the *Tmem232* knockout mice. Exons 4 to 8 of the *Tmem232* locus were chosen as the target site for deletion. (**B**) Morphological analysis showing no obvious differences in the testis or epididymis size between *Tmem232* KO and WT males at 3 months of age. (**C**) Testicular weight of *Tmem232* KO and WT males at 3 months of age. (**D**) Body weights of *Tmem232* KO and WT males at 3 months of age. (**E**) Quantification of testicular weight to body weight ratio of *Tmem232* KO and WT males. (**F**) Mean litter size of *Tmem232* KO together with WT male mice at three months of observation (*n* = 3). Data are represented as mean ± SEM. ns represent not statistically significant, *** *p* < 0.001; Student’s *t* test. (**G**) H&E staining of testicular sections. (**H**) Immunofluorescence staining of anti-α-tubulin (red) antibodies in testes sections.

To determine the in vivo functions of *Tmem232*, CRISPR/Cas9 was employed to generate *Tmem232* KO mice, choosing exons 4 to 8 as target sites for deletion (Figure 1A). Genotyping showed that the founder animals had the intended edit: the *Tmem232* locus in WT male mice is 572 bp, while the locus size in *Tmem232* KO male mice is 424 bp (Figure S1C). We examined adult male mice to identify potential phenotypic changes related to *Tmem232* knockout. There were no major differences found between *Tmem232* KO and WT male mice in testis or epididymis size (Figure 1B), testis weight (Figure 1C), weight of mice (Figure 1D), or the ratio of testis weight to body weight of mice (Figure 1E). Hematoxylin and eosin (H&E) staining (Figure 1G) and immunofluorescence analysis with an antibody targeting flagellum protein α-tubulin (Figure 1H) of testes sections displayed no evident differences in the spermatozoa bound to the testis in the seminiferous tubules of *Tmem232* KO and WT mice.

*Tmem232* KO mice were viable and displayed no apparent abnormalities. Tests on the fertility of mice revealed that, no pups were born from pairings that included *Tmem232* KO male mice (Figure 1F). In contrast, *Tmem232* KO female mice are fertile, with a mean litter size of 7.3 (*n* = 3) (Figure S2B). Additionally, H&E staining of ovary sections exhibited no apparent differences between *Tmem232* KO and WT mice (Figure S2A). Therefore, *Tmem232* deletion induced male sterility, but did not influence the female mice’s fertility.

### 3.3. Tmem232 Knockout Leads to Abnormal Sperm Morphology and Causes Low Sperm Motility

**Figure 2:**
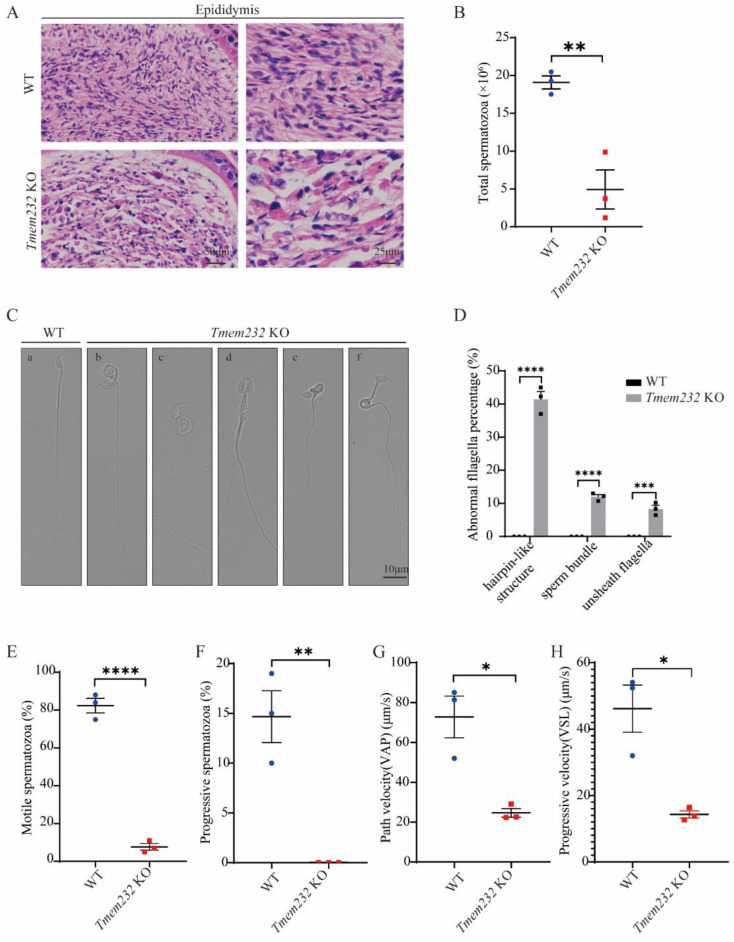
*Tmem232* knockout causes sperm morphological abnormalities and results in spermatozoa with low motility. (**A**) H&E staining of caudal epididymis of *Tmem232* KO and WT male mice. (**B**) Sperm counts in caudal epididymis of *Tmem232* KO and WT male mice. (*n* = 3). Data are expressed as mean ± SEM. ** *p* < 0.01; Student’s *t* test. (**C**) DIC microscopy of epididymal sperm from *Tmem232* KO along with WT mice. (**C**), (**a**) WT mice sperm which had normal sperm head and tail. (**C**), (**b**,**c**,**f**) *Tmem232* KO sperm had hairpin-like structures. (**C**), (**d**) *Tmem232* KO sperm aggregated into bundles. (**C**), (**e**) *Tmem232* KO sperm had hairpin-like structures and had unsheathed flagella. (**D**) Quantification of abnormal spermatozoa for the specified categories. (*n* = 3). Data are represented as mean ± SEM. *** *p* < 0.001, **** *p* < 0.0001; two-way ANOVA test. (**E**) The percent of motile sperm in *Tmem232* KO and WT male mice utilizing a computer-assisted semen analysis system. (*n* = 3). Data are expressed as mean ± SEM.**** *p* < 0.0001; Student’s *t* test. (**F**) The percentage of progressive spermatozoa in *Tmem232* KO and WT male mice. (*n* = 3). Data are expressed as mean ± SEM. ** *p* < 0.01; Student’s *t* test. (**G**,**H**) Mean path velocity and progressive velocity of *Tmem232* KO and WT male mouse spermatozoa (*n* = 3). Data are represented as mean ± SEM. * *p* < 0.05; Student’s *t* test.

To assess sperm head shape and acrosome development during spermatogenesis, we conducted PAS (Periodic Acid Schiff) staining of testes sections. The results showed that the head shape and acrosome development of *Tmem232* KO mice are normal (Figure S3A,B). The number of sperm in the caudal epididymis was quantified through H&E staining which showed that *Tmem232 KO* mice have fewer spermatozoa versus WT mice (Figure 2A,B).

We then used DIC microscopy to observe sperm morphology. The *Tmem232* KO mice had numerous morphologically abnormal sperm in contrast to the sperm of WT mice (Figure 2C): 41.43% of *Tmem232* KO sperm had hairpin-like structures (Figure 2C(b,c,e,f)) in their midpieces, 11.97% of *Tmem232* KO sperm aggregated into bundles (Figure 2C(d)), and 8.31% of *Tmem232* KO sperm had unsheathed flagella (Figure 2C(e)) at their principal piece (Figure 2D). Use a computer-assisted semen analysis system (CASA), we found that compared to WT mice, *Tmem232* KO sperm showed significantly decreased motility. The percentage of motile sperm in WT mice was 82.33%, but in *Tmem232* KO mice it was only 7.667% (Figure 2E). The progressive sperm rate in WT mice was 14.67%, while in *Tmem232* KO mice, no progressive sperm were observed (Figure 2F). The mean path velocity and the straight-line velocity are two essential parameters in evaluating the motility of sperm [13]. We noted that the mean path velocity (Figure 2G) together with straight-line velocity (Figure 2H) significant decreased in sperm of *Tmem232* KO mice compared to WT mice. Thus, knockout of the *Tmem232* resulted in low motility and severe morphological defects in sperm.

### 3.4. Knockdown of Tmem232 Leads to Failed Cytoplasmic Clearance

**Figure 3:**
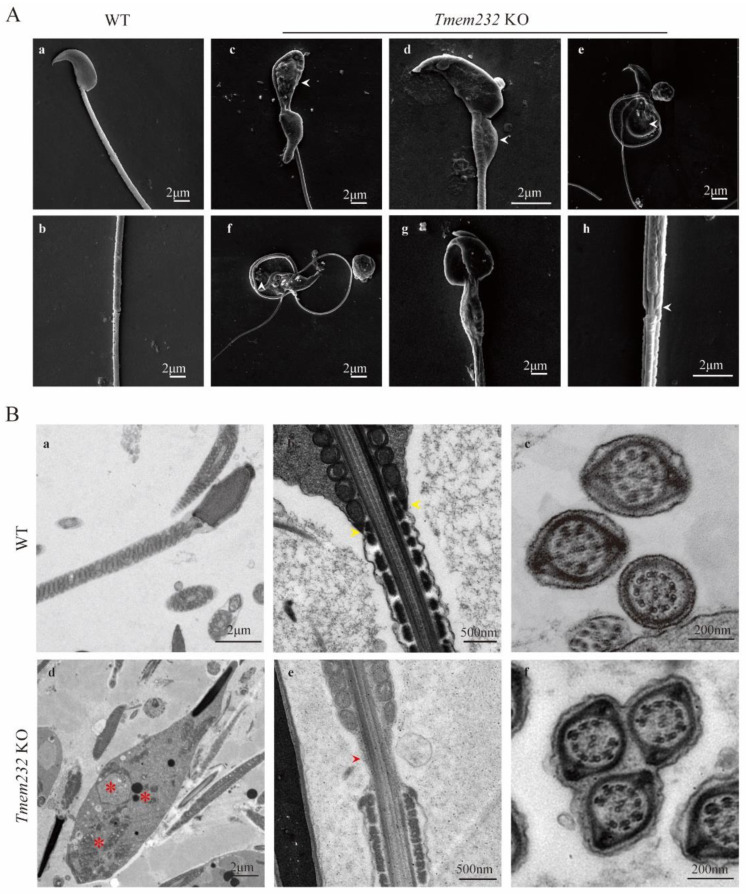
*Tmem232* knockout causes failure of the cytoplasm removal. (**A**) Scanning electron microscopy (SEM) analyses of epididymal spermatozoa of WT and *Tmem232* KO mice. WT sperm with normal head and smooth flagella (**A**), (**a**). WT sperm with a tight connection between the midpiece and principal piece (**A**), (**b**). KO mouse sperm with a large vesicle at the midpiece (**A**), (**c**–**g**), white arrowhead; sperm aggregated into bundles (**A**), (**g**,**h**); sperm with unsheathed flagella at the junction of principal and midpiece piece (**A**), (**h**), white arrowhead. (**B**) Transmission electron microscopy (TEM) analyses of epididymal spermatozoa flagella longitudinal section of WT and *Tmem232* KO sperm. WT sperm showed canonical helical mitochondrial sheaths (**B**), (**a**), and a tightly connected midpiece and principal piece (**B**), (**b**), yellow arrowhead. No sperm bundles were observed in WT mice (**B**), (**c**). The abnormal vesicles observed in *Tmem232* KO mice displayed several electron-dense materials, disorganized mitochondria, and a single, large vacuole (**B**), (**d**), red asterisk. The flagellar longitudinal sections of *Tmem232* KO mouse sperm revealed unsheathed flagella at the junction of the principal and midpiece piece (**B**), (**e**), red arrow-head. multiple *Tmem232* KO mice sperm flagella were wrapped in one cell membrane in cross section (**B**), (**f**).

TEM and SEM were applied to explore the sperm morphological abnormalities. SEM analysis for the *Tmem232* KO epididymal sperm displayed several categories of flagellar abnormalities, including sperm with a large vesicle at the midpiece (Figure 3A(c–g)), sperm aggregated into bundles (Figure 3A(g,h)), and sperm with unsheathed flagella at the junction of principal and midpiece piece (Figure 3A(h)), as observed using DIC microscopy. In contrast, WT sperm displayed smooth flagella without any vesicles at the midpiece (Figure 3A(a)) and a tight connection between the midpiece and principal piece (Figure 3A(b)). Additionally, sperm aggregation into bundles was not detected in WT sperm. In addition, SEM analysis revealed the typical falciform shape of the head and the canonical helical mitochondrial sheath in both WT and KO sperm. This suggested that the absence of TMEM232 affects the flagellar morphology but has no significant effect on the sperm head (Figure 3A).

Consistent with the observations from SEM, the TEM analyses of flagellar longitudinal sections of *Tmem232* KO mouse sperm showed a large vesicle at the midpiece. The analyses of these abnormal vesicles showed several electron dense materials, disorganized mitochondria and a single, large vacuole (Figure 3B(d), red asterisk). We suggest that the large vesicle is the result of the abnormal retention of cytoplasmic remnants that should have been shed during spermiogenesis [19], indicating that *Tmem232* KO mice fail to completely remove the excess cytoplasm during spermiogenesis. In addition, TEM analyses of *Tmem232* KO mouse sperm flagellar longitudinal sections showed unsheathed flagella at the junction of principal and midpiece piece (Figure 3B(e), red arrowhead) and several sperm flagella were wrapped in one cell membrane in cross section (Figure 3B(f)). Meanwhile, the TEM images of WT mouse sperm showed canonical helical mitochondrial sheaths without excess cytoplasmic remnants at the midpiece in longitudinal sections of sperm flagella (Figure 3B(a)) [20,21]. The WT sperm also clearly showed the tight connection of the midpiece and principal piece (Figure 3B(b), yellow arrowhead), and no sperm bundles were observed (Figure 3B(c)). Therefore, the results suggested that the *Tmem232* gene is essential for sperm cytoplasm removal.

### 3.5. Tmem232 Knockout Spermatozoa Flagella Lack Microtubule Doublet 7

**Figure 4:**
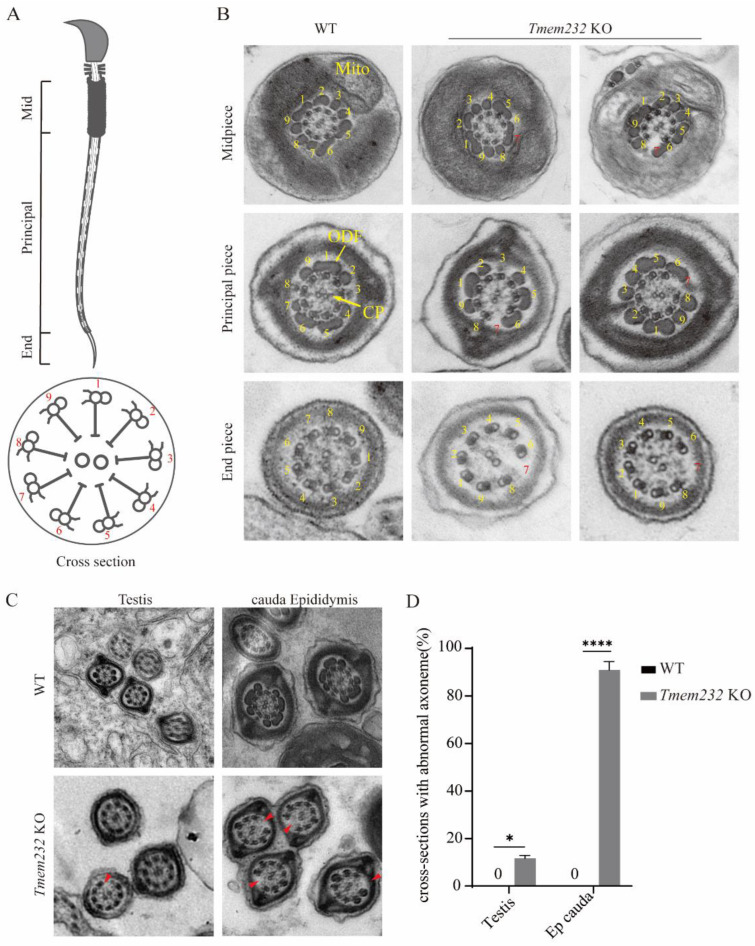
*Tmem232* knockout sperm flagella lack microtubule doublet 7. (**A**) The schematic representation of sperm morphology and its cross-sectional micro-structure composition. (**B**) The ultrastructure of cauda epididymis spermatozoa flagella cross sections by TEM from WT and *Tmem232* KO mice. The sperm flagella of *Tmem232* KO mice reveal absence of the 7th MTD (yellow and red numbers denote MTDs with typical arrangement and missing MTDs, respectively) in the midpiece, principal piece, and end segments of sperm flagella. (**C**) Ultrastructure of flagellar sections with TEM in epididymis cauda and testes from WT along with *Tmem232* KO mice. The sperm flagella of *Tmem232* KO mice reveal absence of the 7th MTD in epididymis cauda and testes (red arrowheads) (**D**) Percentage of flagellar cross-sections with missing 7th MTD in the caudal part of the testis and epididymis (*n* = 3) Data are represented as mean ± SEM. * *p* < 0.05, **** *p* < 0.0001; two-way ANOVA test.

Next, we investigated the effects of *Tmem232* deletion on the flagellar axoneme in mice. When looking at the axoneme from the head of sperm toward the tail, the external MTDs are numbered from 1 to 9. Number 1 is a doublet located in the plane bisecting the microtubule perpendicular to the CP, and number 2 is the next doublet in the clockwise direction (Figure 4A). Cross-sections of the sperm flagella in the caudal epididymis of WT and *Tmem232* KO mice were visualized with TEM. No evident differences were observed in the mitochondrial sheaths of the midpiece and the CP of microtubules in WT and *Tmem232* KO sperm (Figure 4B). However, TEM analysis of the spermatozoa flagella axoneme showed that, the 7th MTD and corresponding ODF in *Tmem232* KO mice was absent compared to the intact flagella axoneme in the WT sperm (Figure 4B).

To further investigate whether the absence of the 7th MTD was related to flagellar biogenesis or destabilization of the 7th MTD [22], the sperm flagella in the testicular seminiferous tubules were analyzed with TEM (Figure 4C). We observed that 11.64% of flagella axonemes had the absence of the 7th MTD in the spermatozoa in the testes of *Tmem232* KO mice, while in 88.77% of flagella axonemes, the absence were observed in caudal epididymis sperm; no absence was observed in WT sperm flagella axonemes. This indicates that *Tmem232* is essential for the maintenance of axoneme integrity.

### 3.6. TMEM232 Interacts with ODF1 and Is Involved in the Formation of ODFs Structure

**Figure 5:**
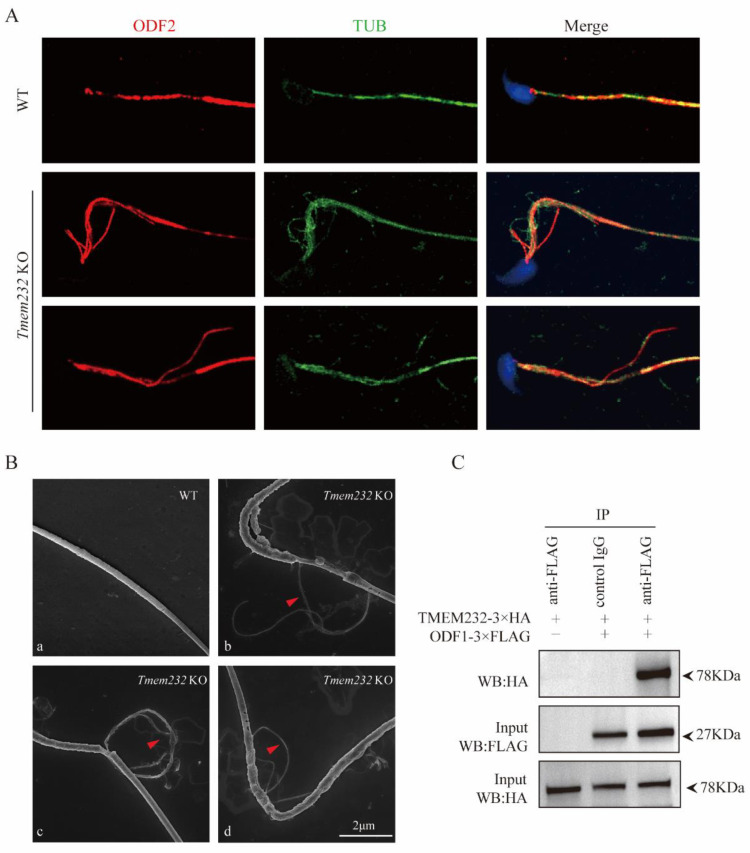
TMEM232 affects the formation of the ODF structures. (**A**) Immunofluorescence staining with antibodies against α-tubulin (green) and ODF2 (red) was performed on individual sperm. (**B**) SEM analyses of epididymal spermatozoa from WT and *Tmem232* KO mice. WT showed normal mitochondrial sheaths and fibrous sheaths (**a**). *Tmem232* KO mice displayed extrusions of ODFs and microtubule doublets ((**b**–**d**) red arrowhead). (**C**) FLAG-*Odf1* and HA-*Tmem232* were co-transfected into HEK293T cells. After 48 h, the cells were gathered for immunoprecipitation with anti-FLAG antibody and examined with FLAG and HA antibodies. The tests were independently conducted thrice with similar results.

Previous studies have identified ODFs as one of the sperm flagella-specific accessory structures surrounding the axoneme in the midpiece to principal piece [3,8,23]. In the absence of microtubules in the axoneme, the corresponding ODFs were also absent [24]. Therefore, we further investigated the relationship between TMEM232 and ODF proteins. We observed the expression of ODFs in individual spermatozoa by performing immunofluorescence including ODF2 and α-tubulin antibodies in *Tmem232* KO and WT mice. The findings showed that the axonemal microtubules and ODFs were extruded from the midpiece and unsheathed flagella at the junction of the principal and midpiece piece in *Tmem232* KO mice, which was not observed in the spermatozoa of WT mice. It is intriguing that despite the pairing of extruded microtubules and ODFs, they are separated externally in the sperm flagella, possibly due to their loose connection and the thinner microtubules’ tendency to break after extrusion (Figure 5A). SEM analyses of epididymal spermatozoa also showed extrusion of ODFs and microtubule doublets at the midpiece and the junction of the principal and midpiece in *Tmem232* KO mice, which was not observed in any of the spermatozoa of WT mice (Figure 5B). These findings suggested that the absence of microtubule doublet 7 and its corresponding ODF in *Tmem232* knockout sperm flagella is due to their extrusion.

To further investigate the relationship between TMEM232 and ODFs, we generated a Flag-tagged *Odf1* plasmid and co-transfected it with a HA-tagged *Tmem232* plasmid into HEK293T cells. Co-immunoprecipitation (co-IP) assays were conducted to study the relationship between the sperm-specific protein ODF1 and TMEM232 in HEK293T cells. The results revealed that TMEM232 could interacted with ODF1 in vitro (Figure 5C). Therefore, our data indicate that TMEM232 binds directly to ODF1 and may act an essential player in sustaining sperm flagellar structure through influencing ODF expression.

## 4. Discussion

To determine the in vivo functions of the testis-enriched and conserved *Tmem232* gene, we generated *Tmem232* KO mice and observed that deficiency of *Tmem232* causes male sterility in mice. The sperm flagellum DIC, SEM and TEM analyses showed an abnormal sperm flagellum morphology in *Tmem232* KO mice. Furthermore, CASA analysis showed that *Tmem232* KO mice had low sperm motility and decreased total sperm count. Through SEM and TEM analyses, we identified that the flagella of *Tmem232* KO sperm had abnormalities in the cytoplasm removal and the flagella axoneme lacked microtubule doublet 7 and the corresponding ODF. Through further phenotypic analysis, we found that the absence of microtubule doublet 7 and its corresponding ODF in *Tmem232* knockout sperm flagella can be attributed to their extrusion from the flagella. Based on the phenotype observed in *Tmem232* KO mice, our study provides the evidence to support the key role of *Tmem232* in the essential processes of cytoplasm removal and maintenance of axoneme integrity.

Efficient removal of cytoplasm is a vital production process for motile spermatozoa. The cytoplasm of germ cells is extruded as the residual body and then engulfed by the mast cells. Nevertheless, the mechanisms of this process remain largely unknown [2,25,26,27]. In our study, *Tmem232* KO mice showed a large vesicle at the midpiece. Using SEM and TEM analyses, we identified this vesicle as the abnormal retention of cytoplasmic remnants that should have been shed during spermiogenesis. According to previous studies, several genes are associated with cytoplasm removal, including *Spem1*, *Ift20*, *Ppp4c*, etc. [2,28,29]. Among them, *Spem1* is also specifically expressed in the testis. Additionally, *Spem1* KO male mice were infertile due to abnormal spermatozoa characterized by a curved head wrapped around the middle portion of the tail and neck. Studies on *Spem1* have indicated that its deficiency causes the cytoplasm to fail to loosen and separate from the neck and head regions of developing sperm. This study demonstrated that appropriate cytoplasmic removal is a genetic regulatory process that requires *Spem1*, and that lack of *Spem1* leads to sperm deformation together with male infertility [2]. In our study, the *Tmem232* KO mice also had abnormal retention of cytoplasmic remnants which was similar to results from *Spem1* KO mice, suggesting that the *Tmem232* gene is essential for sperm cytoplasm removal, which in turn affects the normal formation of sperm structure and consequently impacts male fertility.

ODFs are distinctive accessory structures in the mammalian sperm that serve a function in protecting the sperm tail from shear forces [8]. Currently, there are two predominant types of ODF proteins have been detected in sperm. ODF1 is situated on the ODF surface facing the axoneme, while ODF2 is found in the cortex and is therefore facing the FS of the primary piece and the mitochondria of the midpiece [30]. Both ODF1 and ODF2 not only form a molecular net for ODFs, but they also may interact functionally with various proteins in other tail components [31]. In this work, our findings showed that the axoneme microtubules and ODFs were extruded from the midpiece and unsheathed flagella at the junction of the principal and midpiece piece in *Tmem232* KO mice. In addition, all spermatozoa of *Tmem232* KO mice had an absence of the 7th MTD. This indicates that *Tmem232* serves a vital function in the maintenance of the ODFs’ structure and participates in modulating sperm motility. Research has shown that ODF1 deletion impairs the generation of ODFs, the linkage piece and MS, resulting in sperm head separation and male infertility [3,8]. In our study, co-IP assays were conducted which found that TMEM232 can bind directly to ODF1 in vitro. Therefore, TMEM232 acts a major player in the generation of ODFs by regulating the localization and expression of ODF proteins. This regulatory function directly influences the structural integrity of sperm flagella, which in turn has a significant impact on sperm motility.

## 5. Conclusions

In conclusion, this research provides functional insight into a previously undescribed gene and proved that *Tmem232* is a vital gene that controls normal mouse flagellar structure and sperm motility. As a conserved gene, *Tmem232* has the potential to be a pathogenic gene in clinical azoospermia. Studying the mechanism of *Tmem232* may provide a promising therapeutic target for future infertility research.

## Data Availability

All data are included in the manuscript.

## Supplementary Materials

The following supporting information can be downloaded at: https://www.mdpi.com/article/10.3390/cells12121614/s1, Figure S1: TMEM232 is an evolutionarily conserved testis-enriched protein. (A) Sequence alignment of TMEM232 proteins from several species (mouse, chimpanzee, rat, human, cattle, dog). (B) With Gapdh serving as reference control, RT-PCR of Tmem232 is highly expressed in 3-months testis, but there was weakly expression in brain, liver, lung, spleen, kidney, uterus and epididymis, no expression in heart and ovary; And Tmem232 begin to express in large quantities around postnatal day 21. (C) Genotyping of Tmem232 knockout male mice; Figure S2: Knockout of Tmem232 have no effect on female mice fertility. (A) The hematoxylin staining of WT and Tmem232 KO mice ovary in 3-month (*n* = 3). (B) The average litter size of WT and Tmem232 KO female mice in 3-month (*n* = 3); Figure S3: Sperm nucleus transformation and acrosome formation of Tmem232 KO is normal during spermatogenesis. (A) The PAS and hematoxylin staining of WT and Tmem232 KO mice testes section show. P: pachytene spermatocyte, L: leptotene spermatocyte, Z: zygotene spermatocyte, M: meiotic spermatocyte, rST: round spermatid, eST: elongating spermatid, spz: spermatozoa. (B) The PAS staining of spermatids at different steps from WT and Tmem232 KO mice. From step 1 to step 16 spermatids, the head morphology was normal in Tmem232 KO mice.

## References

[1] de Kretser D.M., Loveland K.L., Meinhardt A., Simorangkir D., Wreford N. (1998). Spermatogenesis. Hum. Reprod..

[2] Zheng H., Stratton C.J., Morozumi K., Jin J., Yanagimachi R., Yan W. (2007). Lack of Spem1 causes aberrant cytoplasm removal, sperm deformation, and male infertility. Proc. Natl. Acad. Sci. USA.

[3] Lehti M.S., Sironen A. (2017). Formation and function of sperm tail structures in association with sperm motility defects. Biol. Reprod..

[4] Miyata H., Morohoshi A., Ikawa M. (2020). Analysis of the sperm flagellar axoneme using gene-modified mice. Exp. Anim..

[5] Eddy E.M., Toshimori K., O’Brien D.A. (2003). Fibrous sheath of mammalian spermatozoa. Microsc. Res. Tech..

[6] Oyama Y., Miyata H., Shimada K., Larasati T., Fujihara Y., Ikawa M. (2022). TULP2 deletion mice exhibit abnormal outer dense fiber structure and male infertility. Reprod. Med. Biol..

[7] Toure A., Martinez G., Kherraf Z.E., Cazin C., Beurois J., Arnoult C., Ray P.F., Coutton C. (2021). The genetic architecture of morphological abnormalities of the sperm tail. Hum. Genet..

[8] Zhao W., Li Z., Ping P., Wang G., Yuan X., Sun F. (2018). Outer dense fibers stabilize the axoneme to maintain sperm motility. J. Cell. Mol. Med..

[9] Gyobu S., Miyata H., Ikawa M., Yamazaki D., Takeshima H., Suzuki J., Nagata S. (2016). A Role of TMEM16E Carrying a Scrambling Domain in Sperm Motility. Mol. Cell. Biol..

[10] Nishimura H., Gupta S., Myles D.G., Primakoff P. (2011). Characterization of mouse sperm TMEM190, a small transmembrane protein with the trefoil domain: Evidence for co-localization with IZUMO1 and complex formation with other sperm proteins. Reproduction.

[11] Noda T., Lu Y., Fujihara Y., Oura S., Koyano T., Kobayashi S., Matzuk M.M., Ikawa M. (2020). Sperm proteins SOF1, TMEM95, and SPACA6 are required for sperm-oocyte fusion in mice. Proc. Natl. Acad. Sci. USA.

[12] Wu B., Yu X., Liu C., Wang L., Huang T., Lu G., Chen Z.J., Li W., Liu H. (2021). Essential Role of CFAP53 in Sperm Flagellum Biogenesis. Front. Cell Dev. Biol..

[13] Huang T., Yin Y., Liu C., Li M., Yu X., Wang X., Zhang H., Muhammad T., Gao F., Li W. (2020). Absence of murine CFAP61 causes male infertility due to multiple morphological abnormalities of the flagella. Sci. Bull..

[14] Xu K., Su X., Fang K., Lv Y., Huang T., Li M., Wang Z., Yin Y., Muhammad T., Liu S. (2023). The Slingshot phosphatase 2 is required for acrosome biogenesis during spermatogenesis in mice. eLife.

[15] Bezerra J.A., da Silva A.M., Peixoto G.C., da Silva Mde A., Franco de Oliveira M., Silva A.R. (2014). Influence of recovery method and centrifugation on epididymal sperm from collared peccaries (*Pecari tajacu* Linnaeus, 1758). Zool. Sci..

[16] Handelsman D.J., Walters K.A., Ly L.P. (2020). Simplified Method to Measure Mouse Fertility. Endocrinology.

[17] Yin Y., Mu W., Yu X., Wang Z., Xu K., Wu X., Cai Y., Zhang M., Lu G., Chan W.Y. (2022). LRRC46 Accumulates at the Midpiece of Sperm Flagella and Is Essential for Spermiogenesis and Male Fertility in Mouse. Int. J. Mol. Sci..

[18] Horowitz E., Zhang Z., Jones B.H., Moss S.B., Ho C., Wood J.R., Wang X., Sammel M.D., Strauss J.F. (2005). Patterns of expression of sperm flagellar genes: Early expression of genes encoding axonemal proteins during the spermatogenic cycle and shared features of promoters of genes encoding central apparatus proteins. Mol. Hum. Reprod..

[19] Hermo L., Pelletier R.M., Cyr D.G., Smith C.E. (2010). Surfing the wave, cycle, life history, and genes/proteins expressed by testicular germ cells. Part 3: Developmental changes in spermatid flagellum and cytoplasmic droplet and interaction of sperm with the zona pellucida and egg plasma membrane. Microsc. Res. Tech..

[20] Sun Q.Y., Liu K., Kikuchi K. (2008). Oocyte-specific knockout: A novel in vivo approach for studying gene functions during folliculogenesis, oocyte maturation, fertilization, and embryogenesis. Biol. Reprod..

[21] Chen Y., Chen X., Zhang H., Sha Y., Meng R., Shao T., Yang X., Jin P., Zhuang Y., Min W. (2022). TBC1D21 is an essential factor for sperm mitochondrial sheath assembly and male fertilitydouble dagger. Biol. Reprod..

[22] Oura S., Kazi S., Savolainen A., Nozawa K., Castaneda J., Yu Z., Miyata H., Matzuk R.M., Hansen J.N., Wachten D. (2020). Cfap97d1 is important for flagellar axoneme maintenance and male mouse fertility. PLoS Genet..

[23] Olson G.E., Sammons D.W. (1980). Structural chemistry of outer dense fibers of rat sperm. Biol. Reprod..

[24] Haidl G., Becker A., Henkel R. (1991). Poor development of outer dense fibers as a major cause of tail abnormalities in the spermatozoa of asthenoteratozoospermic men. Hum. Reprod..

[25] Wang H., Wan H., Li X., Liu W., Chen Q., Wang Y., Yang L., Tang H., Zhang X., Duan E. (2014). Atg7 is required for acrosome biogenesis during spermatogenesis in mice. Cell Res..

[26] Shang Y., Wang H., Jia P., Zhao H., Liu C., Liu W., Song Z., Xu Z., Yang L., Wang Y. (2016). Autophagy regulates spermatid differentiation via degradation of PDLIM1. Autophagy.

[27] Cheng C.Y., Mruk D.D. (2010). The biology of spermatogenesis: The past, present and future. Philos. Trans. R. Soc. Lond. B Biol. Sci..

[28] Han F., Dong M.Z., Lei W.L., Xu Z.L., Gao F., Schatten H., Wang Z.B., Sun X.F., Sun Q.Y. (2021). Oligoasthenoteratospermia and sperm tail bending in PPP4C-deficient mice. Mol. Hum. Reprod..

[29] Zhang Z., Li W., Zhang Y., Zhang L., Teves M.E., Liu H., Strauss J.F., Pazour G.J., Foster J.A., Hess R.A. (2016). Intraflagellar transport protein IFT20 is essential for male fertility and spermiogenesis in mice. Mol. Biol. Cell.

[30] Schalles U., Shao X., van der Hoorn F.A., Oko R. (1998). Developmental Expression of the 84-kDa ODF Sperm Protein: Localization to both the Cortex and Medulla of Outer Dense Fibers and to the Connecting Piece. Dev. Biol..

[31] Shao X., Tarnasky H.A., Lee J.P., Oko R., van der Hoorn F.A. (1999). Spag4, a novel sperm protein, binds outer dense-fiber protein Odf1 and localizes to microtubules of manchette and axoneme. Dev. Biol..

