# Supplementary material for: Deficiency of the Tmem232 Gene Causes Male Infertility with Morphological Abnormalities of the Sperm Flagellum in Mice
Source: Cells. 2023 Jun 13;12(12):1614. doi: 10.3390/cells12121614 (PMC10296807; doi:10.3390/cells12121614)
Supplement: Supplementary file 1 [file cells-12-01614-s001.zip › cells-2389598-supplementary.pdf]

## Supplementary figures

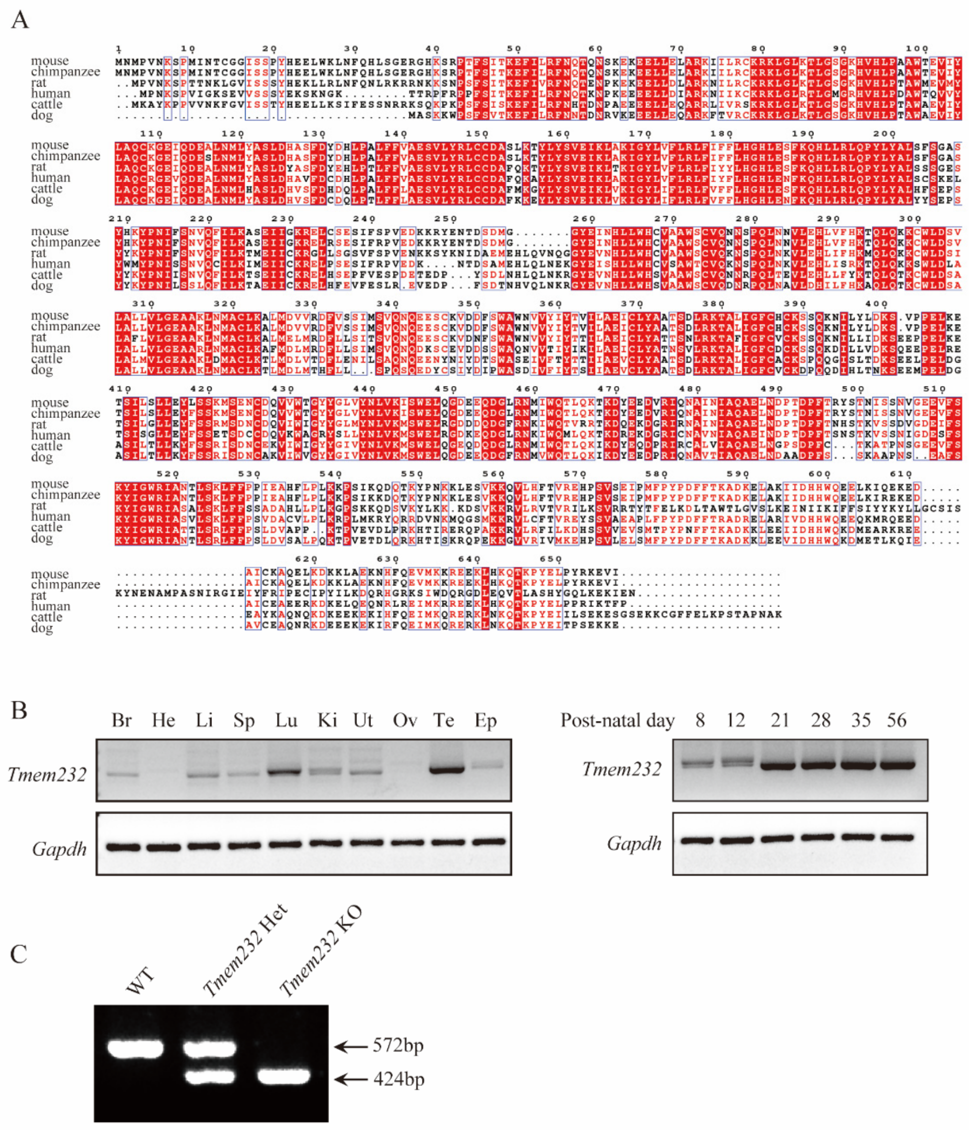


**Figure S1. TMEM232 is an evolutionarily conserved testis-enriched protein.**

**(A)** Sequence alignment of TMEM232 proteins from several species (mouse, chimpanzee, rat, human, cattle, dog).

**(B)** With *Gapdh* serving as reference control, RT-PCR of *Tmem232* is highly expressed in 3-months testis, but there was weakly expression in brain, liver, lung, spleen, kidney, uterus and epididymis, no expression in heart and ovary; And *Tmem232* begin to express in large quantities around postnatal day 21.

**(C)** Genotyping of *Tmem232* knockout male mice.

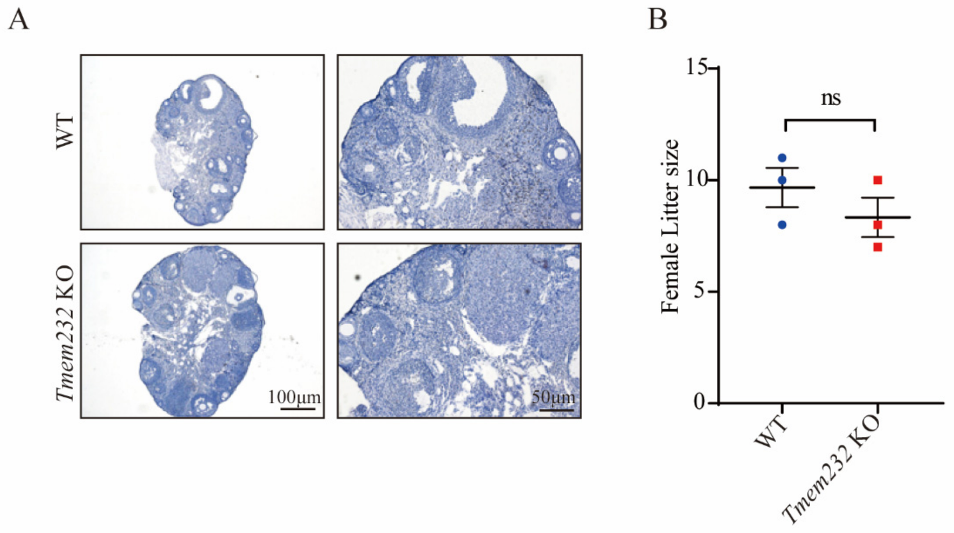


**Figure S2. Knockout of *Tmem232* have no effect on female mice fertility.**

**(A)** The hematoxylin staining of WT and *Tmem232* KO mice ovary in 3-month (n=3).

**(B)** The average litter size of WT and *Tmem232* KO female mice in 3-month (n=3).

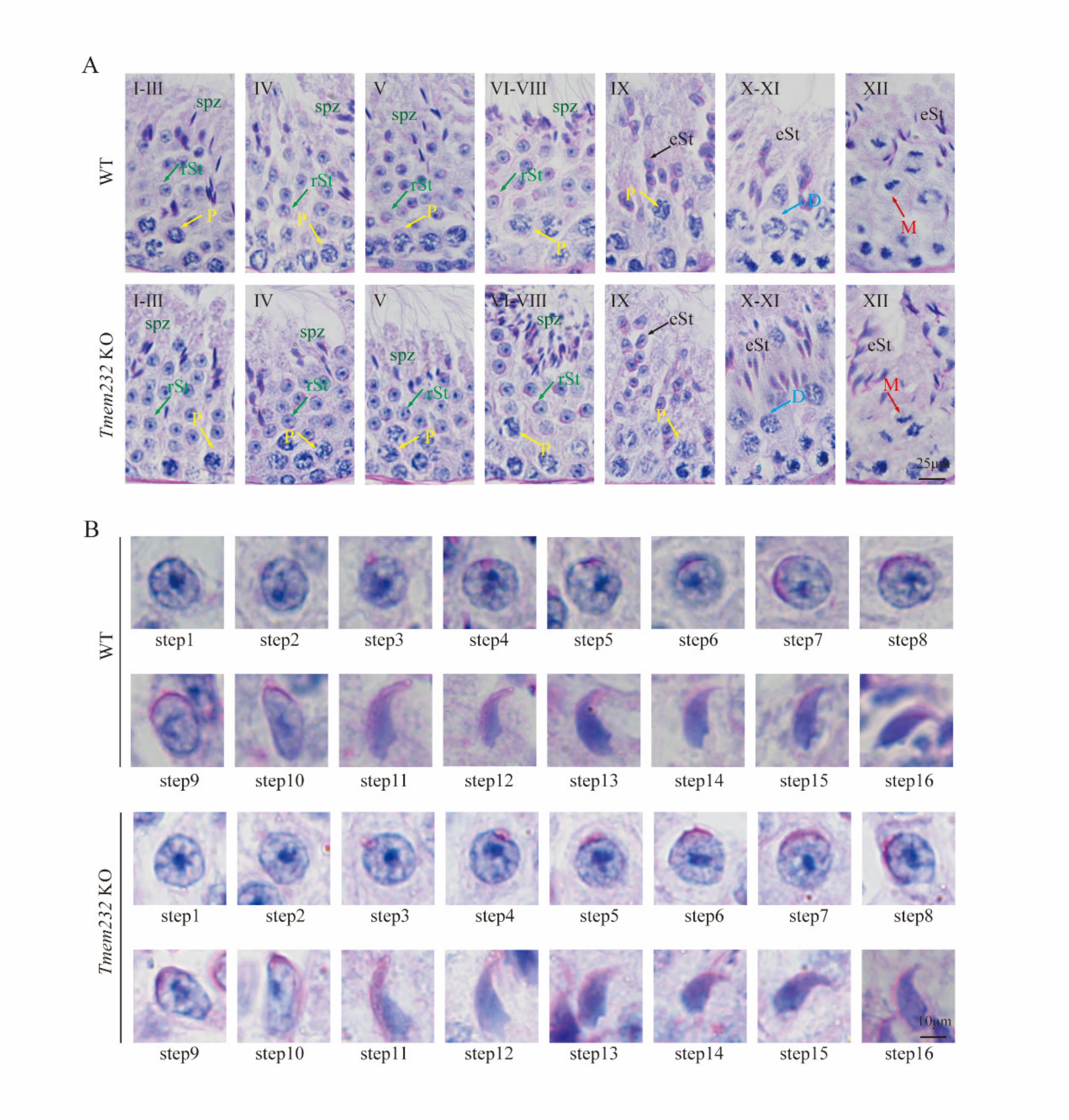


**Figure S3. Sperm nucleus transformation and acrosome formation of *Tmem232 KO* is normal during spermatogenesis.**

**(A)** The PAS and hematoxylin staining of WT and *Tmem232* KO mice testes section show. P: pachytene spermatocyte, L: leptotene spermatocyte, Z: zygotene spermatocyte, M: meiotic spermatocyte, rST: round spermatid, eST: elongating spermatid, spz: spermatozoa.

**(B)** The PAS staining of spermatids at different steps from WT and *Tmem232* KO mice. From step 1 to step 16 spermatids, the head morphology was normal in *Tmem232* KO mice.
